# *Regenerative Medicine Research*: an open access translational medicine journal

**DOI:** 10.1186/2050-490X-1-1

**Published:** 2013-10-01

**Authors:** Y James Kang

**Affiliations:** Director of Regenerative Medicine Research Center, Sichuan University West China Hospital, Chengdu, China; Professor of Pharmacology and Toxicology, University of Louisville School of Medicine, Louisville, Kentucky USA

I am, as an Editor-in-Chief joining BioMed Central, so excited to announce the launch of a new biomedical journal, *Regenerative Medicine Research*. In this open access, online journal, we aim to publish research relating to both the fundamental and practical aspects of regenerative medicine, with a particular emphasis on translational research.

Regenerative medicine is an emerging field with the potential to empower medical research and practice. The process of tissue regeneration is essential to many organisms for a healthy life, from the highly studied axolotl, to us as human beings. In many ways regenerative medicine has become the leading edge of biomedical research and clinical practice, due to its key implications for the treatment of increasingly prevalent diseases, such as heart disease.

The field is in the infant stage of its development but it is growing at an impressively fast pace. For instance, based on a PubMed search, there were less than 200 articles relating to regenerative medicine published in 2002, but more than 3,000 articles published in 2012. However, comprehensive information or research data on regenerative medicine cannot presently be efficiently gathered, but can only be scrutinized through a diversity of different journals in various fields. Regenerative medicine is, by current definition, the process of creating living, functional tissues to repair or replace tissue or organ function lost due to age, disease, damage, or congenital defects [1]. Obviously, this requires a multidisciplinary approach, and one that looks beyond tissue engineering and stem cells. This need calls for a comprehensive journal and therefore, the launch of *Regenerative Medicine Research* takes place.

We hope to publish articles that shed light on the signalling pathways involved in cell/tissue regeneration, as well as those that address how biomaterials and stem cells can be adapted or integrated into the failing organ system. Two of the first articles published in the journal today focus on the effects of chronic alcohol consumption on tissue repair and regeneration; Dekeyser *et al.*[2] study the effect on skeletal muscle and Borrisser-Pairó *et al.*[3] look at levels of IGF-1 myocardial expression. In addition, Coletti *et al*. [4] provide a comprehensive review comparing the cellular mechanisms of skin, nerve and muscle regeneration.

As an open access journal, articles accepted for publication in *Regenerative Medicine Research* are made rapidly available online following acceptance**.** This ensures efficient dissemination of information and experimental data to scientific communities and the medical practice field. Furthermore, a highly respected Editorial Board composed of internationally well-recognized experts in the field facilitates a fair, timely and rigorous peer review process.

The Editorial Board, Publisher, and myself assure the authors and readers that we are committed to making *Regenerative Medicine Research* a preeminent platform for exchanging research protocols and experimental data in this exciting, emerging field. We look forward to receiving your high quality contributions to the journal.
